# Gut Microbiota and Ageing: Mechanisms, Age-Related Diseases, and Therapeutic Perspectives

**DOI:** 10.3390/healthcare14142117

**Published:** 2026-07-15

**Authors:** João Miguel Alves Ferreira, Sergii Tukaiev, Vaitsa Giannouli

**Affiliations:** 1 Institute of Pharmacology and Experimental Therapeutics, Faculty of Medicine, University of Coimbra, 3004-531 Coimbra, Portugal; 2 Coimbra Institute for Clinical and Biomedical Research (iCBR), Faculty of Medicine, University of Coimbra, 3004-531 Coimbra, Portugal; 3 Center for Innovative Biomedicine and Bio-Technology (CIBB), University of Coimbra, 3004-531 Coimbra, Portugal; 4 Faculty of Communication, Culture, and Society, Institute of Public Health, Università Della Svizzera Italiana, 6900 Lugano, Switzerland; tsv.serg.69@gmail.com; 5 Higher Institute of Science Education and Technology, Taras Shevchenko National University of Kyiv, 01033 Kyiv, Ukraine; 6 Department of Psychology, Democritus University of Thrace, 68300 Didymoteicho, Greece

**Keywords:** ageing, gut microbiota, inflammaging, dysbiosis, healthy ageing, frailty, microbiota–gut–brain axis, short-chain fatty acids, Mediterranean diet

## Abstract

Population ageing has intensified interest in biological mechanisms that influence healthspan and susceptibility to age-related diseases. Among these mechanisms, the gut microbiota has emerged as an important modulator of immune, metabolic, and neurophysiological processes involved in ageing. This narrative review critically synthesises current evidence regarding age-related alterations in gut microbiota composition and function, their relationship with inflammaging and the hallmarks of ageing, and the potential role of microbiota-targeted interventions in promoting healthy ageing. A comprehensive narrative literature search was conducted using PubMed, Scopus, and Web of Science, focusing primarily on human observational studies, longitudinal cohorts, mechanistic investigations, and interventional trials published in peer-reviewed journals between 2010 and 2025. Priority was given to studies examining older adults, frailty, longevity, and microbiota-mediated mechanisms relevant to ageing biology. Current evidence suggests that ageing is frequently associated with reduced microbial diversity, depletion of beneficial short-chain fatty acid-producing taxa, altered intestinal barrier integrity, and increased abundance of pro-inflammatory microorganisms. These alterations are linked to inflammaging and may contribute to neurodegenerative, cardiovascular, metabolic, musculoskeletal, and joint diseases, including osteoporosis, sarcopenia, rheumatoid arthritis, and osteoarthritis. However, causality remains incompletely established in humans, and substantial inter-individual variability exists. Studies of centenarians suggest that preservation of specific microbial metabolic functions may be associated with healthier ageing trajectories. Dietary interventions, particularly Mediterranean-style diets rich in fibre and polyphenols, alongside exercise, prebiotics, probiotics, and emerging microbiota-targeted therapies, show potential to modulate these pathways. Despite significant advances, major challenges remain regarding causality, reproducibility, standardisation of microbiome analyses, and personalised therapeutic implementation. Future longitudinal multi-omics studies and precision microbiome interventions will be essential to clarify the translational role of the gut microbiota in healthy ageing and age-related disease prevention.

## 1. Introduction

The phenomenon of population ageing represents one of the most profound demographic transformations of the 21st century. According to United Nations projections, the number of individuals aged 65 years and over is expected to more than double by 2050, with the fastest growth occurring in the oldest-old segment (≥85 years) [1]. This shift carries immense implications for healthcare systems, as advanced age remains the strongest risk factor for multimorbidity, frailty, disability, and mortality from non-communicable diseases [2]. While genetic factors influence approximately 20–30% of lifespan variation, the majority of inter-individual differences in healthspan are attributable to environmental, lifestyle, and stochastic influences [3]. Within this context, the trillions of microorganisms residing in the gastrointestinal tract (collectively termed the gut microbiota) have gained recognition as a pivotal interface between external exposures and host physiology [4,5].

According to the World Health Organization, healthy ageing is defined as the process of developing and maintaining the functional ability that enables well-being in older age [6]. This concept extends beyond the absence of disease and encompasses the preservation of physical, cognitive, psychological, and social functioning, allowing individuals to remain independent and actively engaged in society. As a multidimensional process, healthy ageing results from complex interactions among intrinsic biological factors, lifestyle behaviours, environmental exposures, and social determinants of health. Increasing evidence suggests that the gut microbiota may represent an important mediator of these interactions through its influence on metabolic regulation, immune function, intestinal barrier integrity, and communication along the microbiota–gut–brain axis [4,7].

The human gut harbours a complex ecosystem dominated primarily by bacteria belonging to the phyla Firmicutes, Bacteroidetes, Actinobacteria, and Proteobacteria, alongside archaea, fungi, and viruses. In healthy young adults, this community exhibits high taxonomic and functional diversity, performing essential functions including fermentation of indigestible dietary polysaccharides into short-chain fatty acids (SCFAs), synthesis of vitamins, regulation of bile acid metabolism, competitive exclusion of pathogens, and education of the mucosal immune system [4,8]. These activities extend far beyond the intestine; microbial metabolites and signalling molecules enter systemic circulation, influencing distant organs including the liver, adipose tissue, skeletal muscle, bone, cardiovascular system, and central nervous system through the microbiota–gut–brain axis [7,9,10].

Over the past two decades, culture-independent molecular techniques, particularly 16S rRNA gene sequencing, shotgun metagenomics, and metabolomics, have revolutionised our understanding of how this microbial ecosystem changes across the lifespan. Seminal work by Claesson et al. [11] demonstrated that the gut microbiota of community-dwelling and institutionalised elderly individuals exhibited reduced diversity compared with younger adults, with pronounced inter-individual variability strongly correlated with residence location, diet, and inflammatory status. Subsequent studies across diverse populations have largely corroborated an age-related decline in microbial richness and evenness, although the precise compositional shifts appear context-dependent [8,12,13,14,15].

Research focusing on exceptionally long-lived individuals has provided particularly valuable insights. Biagi et al. [12] compared the faecal microbiota of centenarians, semi-supercentenarians, and younger elderly from Italy, revealing a remodelling characterised by increased abundance of health-associated taxa such as Akkermansia, Christensenellaceae, and butyrate producers, alongside a reduction in potentially pro-inflammatory groups [16]. More recent longitudinal data by Wilmanski et al. [14] demonstrated that unique gut microbiome patterns not only reflect healthy ageing but also have been associated with survival and healthy ageing trajectories independently of several traditional clinical markers.

These age-related microbial alterations are intimately linked to inflammaging, a persistent, sterile, low-grade inflammatory state that constitutes a central hallmark of ageing and drives age-related pathology [17,18]. Inflammaging is mechanistically connected to the broader hallmarks of ageing framework originally proposed by López-Otín et al. [19] and later expanded [20]. Key mechanisms include compromised intestinal barrier integrity leading to translocation of bacterial lipopolysaccharide (LPS) and metabolic endotoxaemia, reduced production of SCFAs, altered secondary bile acid metabolism, and increased generation of pro-inflammatory metabolites such as trimethylamine N-oxide (TMAO) [4,9,21]. These processes interact with mitochondrial dysfunction, cellular senescence, epigenetic alterations, and impaired immune regulation.

Such microbial changes likely contribute to the pathogenesis of multiple age-related diseases. Strong evidence links gut dysbiosis to neurodegenerative disorders, including Alzheimer’s and Parkinson’s disease, through the microbiota–gut–brain axis [7,22,23,24]. Similarly, the microbiota influences cardiovascular ageing via TMAO production [25,26], metabolic deterioration in type 2 diabetes, and musculoskeletal and joint decline, including sarcopenia, osteoporosis, frailty syndrome, rheumatoid arthritis, and osteoarthritis [10,15,27,28].

In contrast, the distinct microbial configurations observed in individuals who reach extreme longevity suggest that the maintenance of specific metabolic functionalities (particularly robust SCFA and secondary bile acid pathways) may likely support healthy ageing rather than merely reflect survival bias [12,14]. Consequently, substantial research has focused on microbiota-targeted longevity strategies. These include Mediterranean and plant-rich dietary patterns [15,29], prebiotics, probiotics, synbiotics, faecal microbiota transplantation [30], regular physical activity [31,32], and pharmacological agents—such as metformin—that exert microbiota-mediated pleiotropic effects [5,21,33].

Several narrative and systematic reviews have explored the relationship between gut microbiota and ageing. However, most have focused either on inflammaging, frailty, neurodegeneration, or microbiota-targeted interventions separately. The present review provides an updated integrative synthesis combining age-related microbial remodelling, mechanisms linking microbiota to hallmarks of ageing, disease-specific evidence, and emerging precision microbiome interventions published up to 2025. Additionally, it critically evaluates causality, methodological limitations, and translational applicability.

This narrative review provides an extensive, critical synthesis of the tripartite relationship between gut microbiota, the ageing process, and age-related diseases, while thoroughly examining the current state of evidence for longevity-promoting microbiota-targeted strategies. By integrating findings from epidemiology, molecular microbiology, geroscience, immunology, and clinical nutrition, it offers a comprehensive framework for understanding how microbial ecology intersects with human ageing trajectories. The review is structured to first delineate age-related microbial changes and underlying mechanisms, subsequently explore disease-specific associations, evaluate interventional approaches, and finally address unresolved challenges and future research priorities. All assertions are grounded exclusively in real, peer-reviewed publications.

### Literature Search Strategy

This narrative review was conducted through a comprehensive literature search using PubMed/MEDLINE, Scopus, Scielo and Web of Science databases. The search focused primarily on peer-reviewed articles published between January 2010 and February 2025, although seminal earlier studies considered foundational to the field were also included where relevant. Search terms included combinations of: “gut microbiota”, “gut microbiome”, “ageing”, “aging”, “inflammaging”, “frailty”, “longevity”, “healthy ageing”, “short-chain fatty acids”, “intestinal permeability”, “leaky gut”, “microbiota–gut–brain axis”, “Alzheimer’s disease”, “Parkinson’s disease”, “cardiovascular disease”, “type 2 diabetes”, “sarcopenia”, “osteoporosis”, “Mediterranean diet”, “probiotics”, “prebiotics”, “synbiotics”, and “faecal microbiota transplantation”. Priority was given to human observational studies, longitudinal cohorts, mechanistic studies, randomised controlled trials, meta-analyses, and high-quality translational investigations involving older adults. Animal studies were included when they provided important mechanistic insights not yet fully established in humans. Studies were selected based on scientific relevance, methodological quality, recency, and contribution to understanding the relationship between gut microbiota and ageing biology. Given the narrative nature of the review, formal systematic review procedures and quantitative meta-analysis were not performed. However, efforts were made to provide a balanced and critical synthesis of the literature, including discussion of conflicting findings, methodological limitations, and challenges regarding causality and reproducibility.

## 2. Age-Related Changes in Gut Microbiota Composition and Function

**Figure 1:**
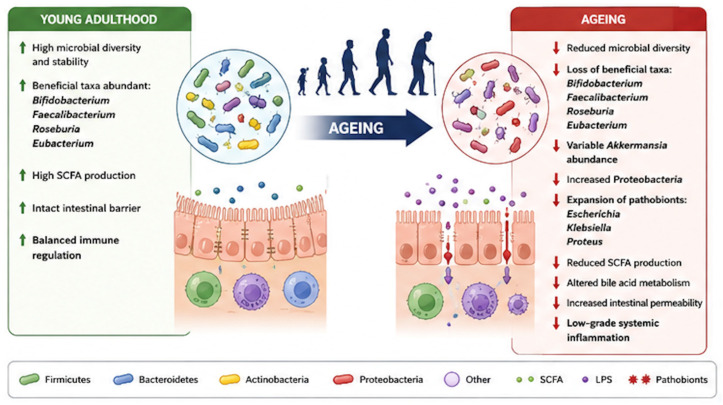
Age-related changes in gut microbiota composition and function.

The compositional and functional alterations that characterise the ageing gut microbiota are summarised in Figure 1. These changes involve progressive reductions in microbial diversity, depletion of beneficial short-chain fatty acid-producing taxa, expansion of opportunistic microorganisms, and alterations in microbial metabolic activity that collectively contribute to inflammaging and age-related physiological decline.

### 2.1. Lifespan Dynamics and General Age-Related Microbial Remodelling

The gut microbiota is not static across the human lifespan but undergoes profound compositional and functional remodelling, with the most clinically significant alterations typically manifesting after the seventh decade of life. While the microbiota of healthy adults is generally characterised by high taxonomic diversity, functional redundancy, and relative stability, ageing introduces a constellation of physiological, immunological, dietary, and environmental factors that collectively erode this equilibrium [4,8]. Culture-independent molecular profiling techniques, particularly 16S rRNA gene amplicon sequencing, shotgun metagenomics, and metabolomic analyses, have provided unprecedented resolution into these changes. Early life is marked by rapid colonisation and succession, progressing from relatively simple communities dominated by Bifidobacterium spp. in breast-fed infants to more complex adult-like configurations by approximately three years of age [34]. Throughout adulthood, the core microbiota remains relatively resilient, although substantial inter-individual variation exists, often clustered into enterotypes dominated by Bacteroides, Prevotella, or Ruminococcus [35]. It is from middle age onwards, and particularly after 65–75 years, that consistent patterns of alteration become evident, although these patterns display considerable heterogeneity influenced by geography, diet, medication use, lifestyle, and frailty status [11,12,14].

A hallmark finding replicated across multiple cohorts is the reduction in alpha diversity (species richness and evenness within an individual sample) in older adults relative to younger controls. The seminal Irish elderly microbiota study by Claesson et al. [11] analysed 178 individuals aged 64–102 years and demonstrated significantly lower microbial diversity in elderly subjects compared with younger adults, with the lowest diversity observed among long-stay residential care residents. This loss of diversity was strongly associated with increased frailty, poorer nutritional status, and inflammatory markers. Community-dwelling elderly showed intermediate profiles, underscoring the dominant role of living environment and diet over chronological age alone. These observations have been broadly corroborated in other European populations [12,13], Asian cohorts [16], and North American studies [27]. Beta diversity analyses (between-sample dissimilarity) further reveal that the elderly microbiota displays greater inter-individual variation than that of younger adults, suggesting a loss of the stabilising forces that maintain community structure in youth [8].

### 2.2. Taxonomic Shifts and Microbial Signatures of Ageing

At the taxonomic level, several consistent shifts have been documented, although the precise genera and species involved can vary by population. There is a frequently reported decline in beneficial, fibre-degrading taxa, particularly certain members of the Firmicutes phylum such as Faecalibacterium prausnitzii, Roseburia spp., Eubacterium spp., and Coprococcus spp., which are major butyrate producers [11,12,36]. Bifidobacterium species, well-known for their health-promoting properties, also tend to decrease in abundance and diversity with advancing age, although probiotic supplementation studies suggest this decline is partially reversible [21,36]. Conversely, there is often an expansion of facultative anaerobes and potential pathobionts belonging to the Proteobacteria phylum, including Escherichia, Klebsiella, and Proteus genera [9,36]. The abundance of Akkermansia muciniphila, a mucin-degrading bacterium associated with improved metabolic health and intestinal barrier integrity, shows conflicting patterns: some studies report its decline with age, while others, particularly in long-lived individuals, document its enrichment [12,14].

### 2.3. Healthy Longevity and Centenarian Microbiota Signatures

Research focusing on exceptionally long-lived individuals has yielded particularly valuable insights, suggesting that successful ageing may be characterised by a distinct microbial configuration rather than simply the avoidance of age-typical dysbiosis. Biagi et al. [12], in their comparison of Italian centenarians (mean age 100 years), semi-supercentenarians (mean age 105 years), young-old (mean age 72 years), and young adults, identified a remodelling of the microbiota in the extreme elderly characterised by an increase in certain health-associated taxa, including Akkermansia, members of the Christensenellaceae family, and several butyrate-producing bacteria, alongside a reduction in certain pro-inflammatory groups. Intriguingly, the microbiota of semi-supercentenarians appeared more similar to that of younger elderly than to “ordinary” centenarians, suggesting that maintenance of specific microbial functionalities may contribute to the ability to reach extreme old age. Kong et al. [16] reported enrichment of specific taxa including members of *Clostridiales*, *Christensenellaceae*, and *Ruminococcaceae* in Chinese centenarians, suggesting potential associations between these microbial signatures and healthy ageing. More recent large-scale longitudinal data from the United States bolster these observations. Wilmanski et al. [14] demonstrated that specific gut microbiome configurations were associated with healthy ageing phenotypes and survival-related outcomes, although causal mechanisms remain incompletely understood. These findings position the gut microbiome not merely as a passive biomarker of chronological age but as an active participant and potential predictor of biological age and longevity.

### 2.4. Functional and Metabolomic Alterations During Ageing

Beyond taxonomic composition, functional capacity of the microbiota, as revealed by shotgun metagenomics and metabolomics, undergoes substantial alteration. There is often a reduction in genes involved in the fermentation of complex polysaccharides and the biosynthesis of SCFAs, particularly butyrate [14,15]. Butyrate is critical for colonic epithelial health, tight junction integrity, histone deacetylase inhibition, and anti-inflammatory signalling via G-protein-coupled receptors (GPR41, GPR43, and GPR109A). Reduced butyrate production is therefore mechanistically linked to compromised barrier function and heightened systemic inflammation. Conversely, pathways associated with the production of pro-inflammatory metabolites, such as lipopolysaccharide (LPS) biosynthesis and trimethylamine (TMA) production (a precursor to the atherogenic TMAO), may be enriched in some elderly cohorts [9,26]). Alterations in bile acid metabolism are also prominent; ageing is associated with reduced microbial conversion of primary to secondary bile acids, which have important signalling roles via the farnesoid X receptor (FXR) and TGR5 receptors that regulate glucose, lipid, and energy homeostasis [21].

### 2.5. Lifestyle, Diet, Medication, and Environmental Drivers

Several factors drive these age-related microbial changes. Dietary patterns in older adults frequently shift towards lower fibre and polyphenol intake and higher consumption of processed foods, directly limiting substrate availability for beneficial saccharolytic bacteria [15,29]. The NU-AGE study, a large European multicentre dietary intervention trial, elegantly demonstrated that one year of Mediterranean diet adherence in older adults (65–79 years) significantly increased microbial diversity, enriched specific fibre-degrading taxa, and reduced frailty scores, with changes in microbiota composition partially mediating improvements in inflammatory status and cognitive function [15]. Polypharmacy is another major driver. Older adults commonly consume five or more medications daily, with proton-pump inhibitors, antibiotics, statins, and metformin exerting particularly strong selective pressures on microbial communities [27,28]. Antibiotics can cause long-lasting reductions in diversity, while non-antibiotic drugs may inadvertently select for specific resistant taxa. Physical activity levels, which typically decline with age, also influence microbial ecology; exercise has been associated with beneficial shifts in microbial composition and increased abundance of health-promoting taxa in older adults [31,32]. Comorbidities such as diabetes, cardiovascular disease, and neurodegenerative conditions further modify the microbiota, creating complex bidirectional relationships [7,10].

### 2.6. Sex-Related Variability in the Ageing Gut Microbiota

Emerging evidence suggests that sex-related biological differences influence gut microbiota composition, immune responses, and ageing trajectories. Hormonal status, body composition, dietary patterns, and immune regulation contribute to sex-specific microbial signatures across the lifespan. Several studies indicate that postmenopausal hormonal changes may influence microbial diversity, intestinal permeability, and inflammatory responses, potentially contributing to differential susceptibility to osteoporosis, cardiovascular disease, and frailty in older women. Conversely, male ageing trajectories may exhibit distinct microbiota-associated metabolic and inflammatory patterns. However, sex-stratified microbiome analyses remain underrepresented in ageing research, and future longitudinal studies incorporating sex-specific analyses will be essential to clarify these interactions [37,38].

### 2.7. Longitudinal Dynamics and Methodological Considerations

Longitudinal studies, though still relatively scarce due to cost and participant retention challenges, have begun to clarify the temporal dynamics of these changes. While cross-sectional designs cannot distinguish cause from consequence, repeated sampling within individuals reveals that microbial trajectories are highly individualised. Some older adults exhibit remarkable microbial stability over years, while others undergo rapid shifts associated with health deterioration, hospitalisation, or antibiotic exposure [13,14]. These longitudinal patterns reinforce the view that the microbiota of the oldest-old is not uniformly “degraded” but may represent an adaptive configuration in those who achieve exceptional longevity.

Methodological considerations are crucial when interpreting this body of work. Faecal sampling, while convenient, primarily reflects distal colonic luminal communities and may under-represent mucosa-associated microbiota, which interact more directly with the host immune system. Variations in DNA extraction protocols, sequencing platforms (Illumina vs. Ion Torrent), 16S hypervariable region targeted, and bioinformatics pipelines (QIIME, mothur, DADA2) can introduce substantial technical bias, complicating direct comparison across studies [39]. Moreover, most studies have focused on Western or East Asian populations, with limited data from Africa, South America, and indigenous communities where dietary and lifestyle contexts differ markedly. Despite these limitations, the cumulative evidence strongly supports the conclusion that the gut microbiota undergoes progressive, diet- and lifestyle-sensitive remodelling with advancing age. These changes are not merely correlative but appear functionally linked to key ageing processes through mechanisms explored in the subsequent section.

### 2.8. Section Synthesis

This section has synthesised a large body of empirical evidence demonstrating that age-related gut microbiota alterations are characterised by reduced diversity, loss of beneficial saccharolytic and butyrate-producing taxa, relative expansion of pathobionts, and functional shifts that impair SCFA production while potentially enhancing pro-inflammatory metabolite generation. These changes are modulated by modifiable factors including diet, medication, and physical activity, and distinct microbial signatures may characterise both frailty and successful extreme longevity [11,12,13,14,15].

## 3. Mechanisms Linking Gut Microbiota to Inflammaging and the Hallmarks of Ageing

Building upon the age-related compositional and functional alterations described in the previous section, increasing evidence suggests that gut microbiota dysbiosis likely contributes to inflammaging and to several interconnected hallmarks of ageing.

**Figure 2:**
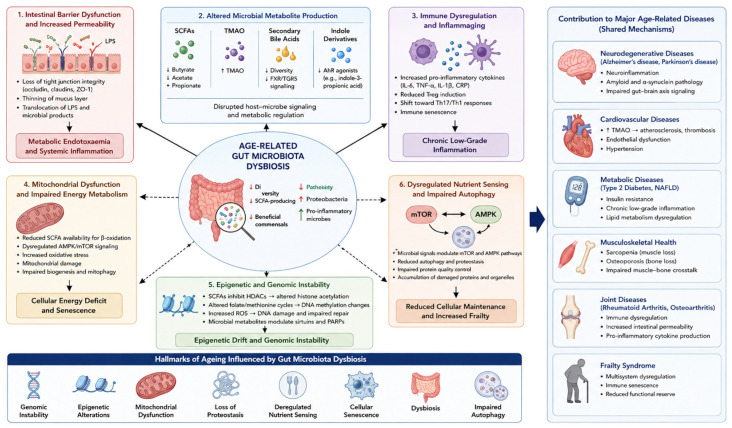
Gut Microbiota and Healthy Ageing: Integrated Mechanistic Framework. The figure illustrates the proposed mechanistic pathways linking age-associated gut microbiota remodelling to inflammaging, immune dysfunction, metabolic impairment, and age-related diseases. Reduced short-chain fatty acid (SCFA) production, increased lipopolysaccharide (LPS) and trimethylamine N-oxide (TMAO) generation, altered bile acid metabolism, and intestinal barrier dysfunction are highlighted as central mechanisms contributing to divergent healthy versus unhealthy ageing trajectories. AD = Alzheimer’s disease; PD = Parkinson’s disease; CVD = cardiovascular disease; T2DM = type 2 diabetes mellitus; RA = rheumatoid arthritis; OA = osteoarthritis.

To integrate the complex bidirectional interactions discussed throughout this review, Figure 2 presents a conceptual framework summarising how ageing, lifestyle factors, diet, medication exposure, and physical activity collectively influence gut microbiota remodelling and downstream biological processes associated with healthy and unhealthy ageing trajectories. The figure synthesises current evidence linking microbial dysbiosis to altered metabolite production, impaired intestinal barrier integrity, inflammaging, immune senescence, metabolic dysfunction, and the development of major age-related diseases.

Inflammaging, a term originally coined by Claudio Franceschi and colleagues, describes the chronic, low-grade, sterile inflammation that characterises ageing organisms and represents a major driver of age-related pathology and frailty [17,18]. Unlike acute inflammatory responses that resolve after the elimination of a pathogen or insult, inflammaging persists and is thought to arise from the cumulative exposure to endogenous and exogenous stressors over decades. Central among these stressors is the progressive remodelling of the gut microbiota, which shifts from a state of symbiotic homeostasis to one of dysbiosis, thereby fuelling systemic inflammatory tone through multiple molecular and cellular pathways [9,10,21]. This section synthesises the mechanistic evidence linking age-associated microbial alterations to inflammaging and to the broader hallmarks of ageing, drawing upon both human observational and interventional data as well as elegant experimental models using gnotobiotic mice, faecal microbiota transplantation (FMT), and targeted microbial manipulations.

Additional mechanistic insights have also emerged from studies using *Caenorhabditis elegans*, a valuable experimental model for investigating conserved host–microbiota interactions involved in lifespan regulation, stress resistance, and nutrient-sensing pathways [40,41]. Due to its short lifespan, genetically tractable biology, and conserved metabolic signalling pathways, *C. elegans* has become increasingly useful for studying microbiota-mediated effects on ageing and host physiology.

**Figure 3:**
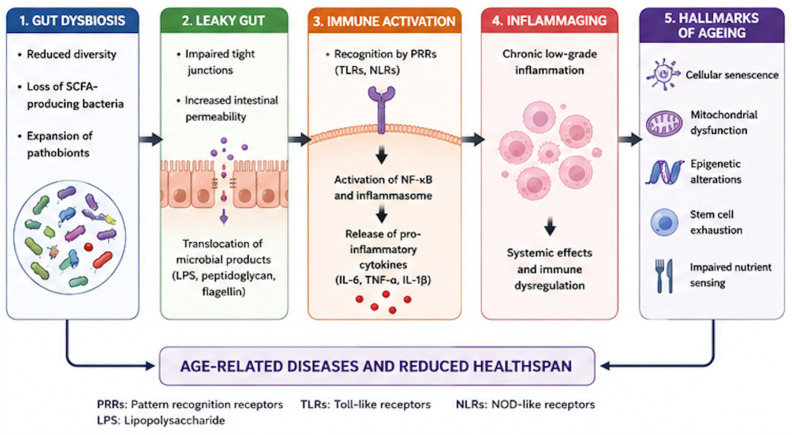
Mechanisms linking gut dysbiosis to inflammaging.

The principal biological pathways through which age-related gut dysbiosis may contribute to inflammaging and the hallmarks of ageing are summarised in Figure 3.

### 3.1. Intestinal Barrier Dysfunction and Leaky Gut in Ageing

Increasing evidence suggests that deterioration of intestinal barrier integrity (“leaky gut”) represents a central mechanistic link between age-related microbiota alterations and inflammaging. Ageing is associated with progressive disruption of epithelial tight junction proteins, including zonula occludens-1, occludin, and claudins, resulting in increased intestinal permeability and translocation of microbial-derived products such as lipopolysaccharide (LPS) into systemic circulation [9,10]. Recent evidence further supports the importance of microbial diversity as a marker of healthy ageing. Ghosh et al. [42] reported that preservation of microbial richness and functional redundancy is associated with greater resilience against age-related physiological decline, reinforcing the concept that microbial diversity may serve as a key indicator of biological ageing trajectories. Emerging evidence also indicates that age-related disruption of tight-junction proteins and mucosal barrier integrity may contribute to systemic low-grade inflammation and metabolic dysfunction, further strengthening the proposed role of intestinal permeability in the ageing process [43].

This process contributes to chronic low-grade systemic inflammation through activation of Toll-like receptor signalling and persistent production of pro-inflammatory cytokines including tumour necrosis factor-α (TNF-α), interleukin-6 (IL-6), and C-reactive protein (CRP). Importantly, lifelong exposure to inflammatory insults, poor dietary quality, reduced fibre intake, infections, and repeated antibiotic exposure may progressively impair epithelial repair mechanisms and accelerate barrier dysfunction during ageing [17,21].

Experimental evidence from germ-free mouse models has further highlighted the importance of intestinal permeability in ageing biology. Thevaranjan et al. [9] demonstrated that aged germ-free mice exhibited reduced systemic inflammation and preserved intestinal barrier integrity compared with conventionally colonised aged mice, suggesting that microbiota-driven inflammatory signalling contributes substantially to age-associated inflammaging. These findings also indicate that microbial communities are not universally beneficial but instead exist along a spectrum ranging from protective and symbiotic to potentially pro-inflammatory depending on ecological context, host resilience, and environmental exposures.

Conversely, dietary fibre, Mediterranean-style dietary patterns, regular physical activity, and maintenance of microbial diversity throughout life appear to support barrier integrity by enhancing short-chain fatty acid production, particularly butyrate, which promotes epithelial repair and tight junction stability [4,15].

### 3.2. Microbial Metabolites, Nutrient Sensing, and Ageing Pathways

Microbial metabolites serve as key signalling molecules that translate luminal ecology into host physiological responses. Short-chain fatty acids (SCFAs), particularly butyrate, propionate, and acetate, produced through the fermentation of dietary fibre by taxa such as *Faecalibacterium prausnitzii*, *Roseburia* spp., *Eubacterium rectale*, and certain *Bifidobacterium* species, exert potent anti-inflammatory and barrier-strengthening effects [4,15]. Butyrate functions as a histone deacetylase (HDAC) inhibitor, epigenetically modulating gene expression in both epithelial and immune cells to promote regulatory T-cell differentiation, suppress NF-κB signalling, and enhance tight junction assembly. It also activates G-protein-coupled receptors (GPR41/FFAR3, GPR43/FFAR2, and GPR109A/HCAR2), which regulate energy metabolism, insulin sensitivity, and anti-inflammatory programmes. Age-related depletion of these SCFA producers, documented across multiple cohorts, results in lower faecal and circulating SCFA concentrations, thereby potentially contributing to reduced anti-inflammatory regulation during ageing [12,14]. In the NU-AGE Mediterranean diet intervention involving older adults across five European countries, increased intake of fibre and polyphenols led to enrichment of SCFA-producing taxa, elevated SCFA levels, reduced inflammatory markers, and decreased frailty scores, with microbiota changes statistically mediating improvements in health status [15].

Conversely, certain microbial metabolites become detrimental with age. The conversion of dietary choline and L-carnitine into trimethylamine (TMA) by enzymes encoded by the *cutC/D* gene cluster in certain Proteobacteria and Firmicutes, followed by hepatic oxidation to trimethylamine N-oxide (TMAO), represents a metabolic pathway that has been associated with age-related cardiometabolic dysfunction [25,26]. Elevated TMAO promotes endothelial dysfunction, platelet hyperreactivity, atherosclerosis, and renal fibrosis, all of which are amplified in older individuals. Similarly, altered bile acid metabolism, characterised by reduced microbial 7α-dehydroxylation activity that converts primary bile acids into secondary forms (such as deoxycholic and lithocholic acids), disrupts signalling through farnesoid X receptor (FXR) and Takeda G-protein receptor 5 (TGR5). These receptors regulate lipid and glucose homeostasis; their dysregulation contributes to metabolic inflammation [21]. Indole derivatives produced from tryptophan by Lactobacillus and Clostridium species can be protective via the aryl hydrocarbon receptor (AhR), but their production often declines with age-related microbial shifts.

These metabolite-driven processes intersect directly with the hallmarks of ageing originally delineated by López-Otín et al. [19] and later expanded into an “ageing universe” that includes disabled macroautophagy, chronic inflammation, and dysbiosis itself as an integrative hallmark [20]. Microbial influence on genomic instability is evidenced by SCFA-mediated HDAC inhibition, which can modulate DNA repair pathways, while chronic LPS exposure increases reactive oxygen species (ROS) production, exacerbating oxidative damage to nuclear and mitochondrial DNA. Epigenetic alterations are profoundly affected; butyrate’s HDAC inhibitory activity influences histone acetylation landscapes, while microbial-derived folate and methionine cycle intermediates affect DNA methylation patterns that drift with age [21,44]. Mitochondrial dysfunction and dysregulated nutrient sensing are tightly linked to microbial signals. SCFAs serve as substrates for β-oxidation and influence AMPK and mTOR pathways, two master regulators of cellular metabolism whose dysregulation is central to ageing. Reduced butyrate availability in older adults may therefore promote mitochondrial inefficiency and inappropriate mTOR activation, accelerating cellular senescence [44]. The mechanistic target of rapamycin (mTOR) pathway represents another major interface between gut microbiota-derived metabolites and ageing biology. mTOR signalling regulates autophagy, nutrient sensing, mitochondrial metabolism, protein synthesis, and immune function, all of which are central hallmarks of ageing [20]. Short-chain fatty acids, amino acid availability, bile acid signalling, and microbial-derived metabolites may influence mTOR activity directly or indirectly through AMPK and insulin signalling pathways. Dysregulated mTOR activation has been associated with inflammaging, impaired autophagy, anabolic resistance, and immune senescence in older adults. Emerging evidence further suggests bidirectional interactions whereby microbiota composition may modulate mTOR activity while host nutrient-sensing pathways simultaneously shape microbial ecology. These findings reinforce the concept that microbiota–ageing interactions occur within a broader immunometabolic network rather than through isolated inflammatory mechanisms alone [21,44].

### 3.3. Hallmarks of Ageing and Cellular Senescence

Cellular senescence represents another important interface between gut microbiota dysbiosis and ageing biology. Senescent cells secrete a senescence-associated secretory phenotype (SASP) rich in IL-6, IL-1β, and matrix metalloproteinases that may further reshape the intestinal environment, favouring the expansion of pro-inflammatory pathobionts and reinforcing chronic inflammatory signalling [17]. Stem cell exhaustion, particularly of intestinal stem cells and haematopoietic stem cells, is modulated by microbial influences on Wnt and Notch signalling, with butyrate playing a dual role depending on concentration. Altered intercellular communication is perhaps the most directly affected hallmark, as inflammaging represents a paradigmatic example of disrupted signalling networks driven by microbial products [9]. Recent mechanistic evidence suggests that age-related alterations in microbial communities may affect both innate and adaptive immune pathways through changes in microbial metabolites, epithelial barrier signalling, and host–microbe immune interactions [45]. These findings further support the bidirectional relationship between gut microbiota composition and immune ageing.

### 3.4. Gut–Brain Axis and Immune Senescence

The microbiota–gut–brain axis provides an additional layer of mechanistic complexity linking gut dysbiosis with neuroinflammation and cognitive ageing. Vagal nerve signalling, microbial modulation of tryptophan metabolism into kynurenine versus serotonin pathways, and direct effects of short-chain fatty acids on microglial activity appear to connect intestinal microbial ecology with central nervous system function [7].

Experimental studies in aged murine models have shown that transplantation of dysbiotic microbiota is associated with increased brain IL-1β and TNF-α expression, whereas restoration of Akkermansia or butyrate-producing taxa may attenuate these neuroinflammatory responses [7,22]. Immune senescence may also be exacerbated by microbial-driven shifts in T-cell polarisation, with reduced short-chain fatty acid signalling impairing the differentiation of anti-inflammatory regulatory T cells while promoting pro-inflammatory Th17 and Th1 responses [10].

In addition to murine models, the nematode *Caenorhabditis elegans* has emerged as an important experimental model for investigating microbiota–ageing interactions due to its short lifespan, conserved nutrient-sensing pathways, and tractable host–microbe interactions. Studies using *C. elegans* have demonstrated that specific microbial strains and microbial metabolites can influence lifespan, stress resistance, immune signalling, and mitochondrial function, supporting the broader concept that host–microbiota interactions modulate ageing biology across species [40,41].

### 3.5. Translational Evidence, Limitations, and Future Directions

Longitudinal and multi-omics studies have begun to establish temporal and mechanistic relationships between gut microbiota configurations and healthy ageing trajectories. Wilmanski et al. [14] reported that gut microbiome uniqueness was associated with healthier ageing trajectories and survival-related outcomes in community-dwelling adults. However, the study was observational and did not establish direct mechanistic links between microbiome composition and mitochondrial or immune function. These findings align with experimental evidence suggesting that caloric restriction and metformin treatment may partially exert beneficial effects through microbiota remodelling and modulation of inflammatory pathways [5,33].

Nevertheless, important caveats remain. Causality remains difficult to establish unequivocally in humans due to the bidirectional nature of host–microbe interactions; inflammation itself may reshape the microbiota, thereby generating self-reinforcing pathological cycles. Most mechanistic insights derive from rodent models whose microbiota composition and immune systems differ substantially from humans. Inter-individual variability driven by genetics, geography, medication exposure, dietary patterns, and lifelong environmental influences further complicates generalisation of findings [39].

Technical limitations in metabolomic coverage, sequencing standardisation, and the challenge of distinguishing microbial-derived from host-derived metabolites also persist. Despite these limitations, the convergence of evidence from observational studies, mechanistic animal models, and emerging human interventional data supports the view that age-related gut microbiota dysbiosis likely contributes to inflammaging and to the progression of multiple ageing-related pathological processes.

## 4. The Contribution of Gut Microbiota to Specific Age-Related Diseases

The age-associated remodelling of the gut microbiota, characterised by reduced diversity, depletion of beneficial SCFA-producing taxa, and relative enrichment of pathobionts, does not merely correlate with the ageing process but may also contribute to the pathogenesis and progression of multiple age-related diseases. This contribution occurs through the same mechanisms previously outlined (compromised intestinal barrier function, metabolic endotoxaemia, altered metabolite profiles, and persistent inflammaging) that intersect with tissue-specific vulnerabilities in the ageing host [9,10,17]. Rather than representing a uniform dysbiotic signature, the microbiota appears to exert disease-specific effects modulated by host genetics, lifelong exposures, and concurrent comorbidities. This section provides an extensive synthesis of the evidence linking gut microbial ecology to major age-related conditions, including neurodegenerative disorders, cardiovascular disease, type 2 diabetes mellitus, musculoskeletal frailty, sarcopenia, osteoporosis, and the frailty syndrome itself. All interpretations are grounded in peer-reviewed human observational and longitudinal studies, interventional trials, and mechanistic experiments using gnotobiotic animal models and faecal microbiota transplantation (FMT).

### 4.1. Neurodegenerative Diseases and the Microbiota–Gut–Brain Axis

The strongest and most mechanistically developed evidence concerns the microbiota–gut–brain axis in neurodegenerative diseases, particularly Alzheimer’s disease (AD) and Parkinson’s disease (PD). Cryan et al. [7], in their comprehensive review of the axis, synthesised how microbial signals influence brain function and pathology via neural (vagus nerve), endocrine (hypothalamic–pituitary–adrenal axis), and immune pathways. In PD, Sampson et al. [22] provided landmark causal evidence by demonstrating that germ-free mice or those treated with antibiotics were protected from motor deficits and neuroinflammation when injected with α-synuclein preformed fibrils. Transplantation of microbiota from PD patients into germ-free mice exacerbated motor dysfunction and neuroinflammation compared with transplants from healthy controls, directly implicating specific microbial configurations in disease modulation. Human studies have consistently reported reduced abundance of SCFA producers such as *Faecalibacterium* and *Roseburia* alongside increased Proteobacteria in PD patients, with these shifts correlating with disease severity and gastrointestinal symptoms that often precede motor manifestations by decades [7,10].

In Alzheimer’s disease, similar patterns emerge with additional emphasis on amyloid and tau pathology. Cross-sectional studies of AD patients reveal lower microbial diversity, reduced Bifidobacterium and Eubacterium, and increased abundance of pro-inflammatory taxa such as Escherichia/Shigella, which correlate with cerebrospinal fluid biomarkers of amyloid and tau pathology as well as peripheral inflammation [23,24]. Mechanistic work in transgenic AD mouse models has shown that antibiotic-induced microbiota depletion or germ-free conditions can reduce amyloid plaque deposition and microglial activation, while recolonisation with specific taxa can either ameliorate or exacerbate pathology depending on the community composition [46]. Microbial metabolites appear central: reduced butyrate production may impair histone acetylation and microglial clearance of amyloid, while elevated LPS and TMAO can cross a compromised blood–brain barrier to promote neuroinflammation [7,9]. Longitudinal data from cohorts such as the Rush Memory and Aging Project have linked baseline microbial diversity and specific taxa to subsequent cognitive decline rates, suggesting the microbiota may serve as both a risk marker and modifiable contributor to AD progression in older adults [24]. These findings are particularly relevant given that gastrointestinal dysfunction and systemic inflammation often precede cognitive symptoms, positioning the gut microbiota as a potential early therapeutic target in the long preclinical phase of neurodegenerative diseases.

### 4.2. Cardiovascular and Metabolic Diseases

Cardiovascular diseases (CVD), the leading cause of death in individuals over 65 years, are similarly influenced by microbial metabolism. The seminal work of the Hazen laboratory established that gut microbial conversion of dietary phosphatidylcholine and L-carnitine into trimethylamine (TMA), subsequently oxidised in the liver to trimethylamine N-oxide (TMAO), promotes atherosclerosis, platelet hyperreactivity, and thrombosis [25,26]. Circulating TMAO levels rise with age and strongly predict major adverse cardiovascular events independent of traditional risk factors. In older adults, the age-associated expansion of TMA-producing taxa (certain Clostridium and Proteobacteria species) combined with reduced SCFA producers creates a pro-atherogenic metabolic milieu [21,26]. Secondary bile acid signalling is also perturbed; reduced microbial conversion to anti-inflammatory secondary bile acids impairs FXR and TGR5 signalling, contributing to dyslipidaemia and vascular stiffness [21]. Human studies in elderly cohorts have linked lower microbial diversity and butyrate production capacity with increased arterial stiffness and carotid intima-media thickness, even after adjustment for diet and medication [10]. Interventional evidence further supports causality: dietary interventions that reduce TMAO precursors or supplementation with specific fibre types can lower TMAO and inflammatory markers in older adults [4].

Metabolic diseases, particularly type 2 diabetes mellitus (T2DM) and its complications in the context of ageing, demonstrate clear microbiota involvement. Age-related insulin resistance is exacerbated by metabolic endotoxaemia and reduced SCFA signalling, both of which impair insulin signalling pathways in skeletal muscle, liver, and adipose tissue [21,47]. Shotgun metagenomic studies have identified reduced butyrate biosynthesis genes and increased oxidative stress-related pathways in the microbiota of individuals with T2DM, patterns that overlap substantially with those observed in frail older adults [14,48]. In older populations, polypharmacy (particularly metformin) interacts with the microbiota; while metformin itself remodels the microbiome towards increased Akkermansia and SCFA producers, chronic use in the context of advanced age may select for distinct configurations [33]. Longitudinal data indicate that baseline microbial signatures predict incident metabolic deterioration in older adults better than some clinical parameters, reinforcing the microbiota’s role in age-associated metabolic decline [14].

### 4.3. Frailty, Sarcopenia, and Musculoskeletal Ageing

The gut-muscle axis and its relationship to sarcopenia, osteoporosis, and the frailty syndrome have gained substantial attention in geroscience. Ticinesi et al. [27] proposed the existence of a gut-muscle axis whereby microbial metabolites influence muscle protein synthesis, mitochondrial function, and anabolic resistance in older adults. Frail individuals consistently exhibit lower alpha diversity, reduced Faecalibacterium and Bifidobacterium, and higher Proteobacteria and Bacteroidetes compared with age-matched non-frail controls [10,28]. These compositional shifts correlate with lower grip strength, gait speed, and physical performance scores. Mechanistically, reduced butyrate and other SCFAs may impair muscle mitochondrial biogenesis and promote anabolic resistance via effects on PGC-1α and mTOR signalling. Sarcopenia and frailty are further amplified by inflammaging driven by translocated microbial products [9,10]. The NU-AGE trial provided causal-interventional support: one year of Mediterranean diet intervention in older adults improved frailty status partly through microbiota-mediated increases in SCFA producers and reductions in inflammatory markers [15].

Osteoporosis, another major age-related musculoskeletal disorder, is linked to the microbiota through effects on calcium absorption, immune regulation of osteoclastogenesis, and systemic inflammation. Microbial SCFAs enhance calcium uptake and suppress RANKL-induced osteoclast differentiation, while dysbiosis-driven inflammation promotes bone resorption [21,44]. Human studies have reported associations between low microbial diversity, reduced SCFA producers, and lower bone mineral density in postmenopausal women and older men, although longitudinal data remain limited. FMT experiments in germ-free mice have shown that transplantation of dysbiotic “aged” microbiota can impair bone mass accrual, suggesting directionality [49]. Emerging evidence further indicates that gut microbiota dysbiosis may also contribute to age-related joint diseases, particularly rheumatoid arthritis and osteoarthritis [49,50,51]. Altered intestinal permeability, chronic low-grade inflammation, immune dysregulation, and reduced production of short-chain fatty acids have been implicated in promoting synovial inflammation, cartilage degradation, and abnormal bone remodelling through the gut–joint axis. Although the evidence is currently stronger for rheumatoid arthritis than for osteoarthritis, growing experimental and clinical data suggest that microbial composition and microbial-derived metabolites may influence disease onset, severity, and progression [49,50,51]. Nevertheless, additional longitudinal and mechanistic human studies are required to establish causality and determine whether microbiota-targeted interventions can meaningfully modify joint disease outcomes in older adults.

### 4.4. Additional Age-Related Conditions and Multimorbidity

Beyond these major conditions, emerging evidence links gut microbiota alterations to other age-related pathologies, including chronic kidney disease (via the gut-kidney axis and increased production of uraemic toxins such as indoxyl sulphate and p-cresyl sulphate by altered taxa), certain cancers (particularly colorectal cancer, where age-associated pathobionts may promote genotoxicity and chronic inflammation), and multimorbidity itself [4,14]. The concept of “inflammaging-driven multimorbidity” is increasingly viewed through a microbial lens, whereby a core set of microbial features (low diversity, low SCFA capacity, high LPS/TMAO potential) contributes to the simultaneous deterioration of multiple organ systems [17].

### 4.5. Healthy Ageing and Longevity Signatures

Importantly, studies of centenarians and semi-supercentenarians provide a contrasting perspective. Biagi et al. [12] and Wilmanski et al. [14] demonstrated that exceptionally long-lived individuals often harbour microbiota configurations that avoid the most deleterious features of typical age-related dysbiosis. This suggests that disease-associated microbial signatures are not inevitable consequences of chronological age but modifiable features that distinguish unhealthy from successful ageing.

### 4.6. Biomarker Potential, Clinical Translation, and Reproducibility Challenges

The growing association between gut microbiota signatures and age-related diseases has generated substantial interest in the potential use of microbial profiles as diagnostic, prognostic, and therapeutic biomarkers. Several studies have suggested that specific microbial taxa, metabolite patterns, and functional pathways may help identify individuals at increased risk of frailty, cognitive decline, cardiovascular disease, metabolic dysfunction, and unhealthy ageing trajectories. For example, elevated trimethylamine N-oxide (TMAO), reduced short-chain fatty acid-producing taxa, and decreased microbial diversity have repeatedly been associated with adverse ageing phenotypes and systemic inflammation. However, despite these promising findings, important translational limitations remain. Disease-associated microbiome signatures often demonstrate poor reproducibility across cohorts due to substantial heterogeneity in diet, geography, ethnicity, medication exposure, sequencing methodologies, analytical pipelines, and host genetics. Inter-individual variability further complicates the establishment of universal microbial biomarkers. Additionally, many observed microbial alterations are not disease-specific and may instead reflect broader inflammatory or metabolic disturbances associated with ageing and multimorbidity. Current evidence therefore supports cautious interpretation of microbiome-based diagnostic approaches. While microbiota-guided patient stratification and personalised microbiome interventions represent promising future directions, substantial methodological standardisation, longitudinal validation, and integration of multi-omics datasets will be required before routine clinical implementation becomes feasible [14,39].

### 4.7. Critical Appraisal and Current Limitations

Critical interpretation of this literature reveals both strengths and limitations. The convergence of human associative data, animal mechanistic studies using FMT, and emerging interventional trials provides robust support for microbial contributions to disease. However, many studies remain cross-sectional, suffer from confounding by diet, medication, and comorbidities, and lack standardisation in microbiome methodology [8,39]. Furthermore, substantial heterogeneity in sequencing methodologies, bioinformatic pipelines, dietary assessment tools, and population characteristics continues to limit reproducibility across microbiome studies. Causality is more firmly established in neurodegenerative and metabolic models than in musculoskeletal disease. An additional challenge concerns the interpretation of age-associated microbial signatures themselves. While reduced diversity and depletion of SCFA-producing taxa are frequently described as hallmarks of gut dysbiosis, evidence from centenarian cohorts suggests that healthy ageing may not be characterised by the preservation of a youthful microbiota, but rather by the emergence of adaptive microbial configurations that maintain key metabolic functions despite taxonomic remodelling [12,14]. This distinction is important because it suggests that future therapeutic strategies should prioritise restoration of microbial functionality rather than attempting to recreate a specific taxonomic composition. Consequently, functional and metabolomic profiling may ultimately prove more informative than taxonomic analyses alone when assessing microbiome contributions to healthy ageing and longevity.

Nevertheless, the cumulative evidence positions the gut microbiota as a central node in the complex network linking ageing biology to clinical disease manifestation in older adults. This framework naturally leads to the exploration of therapeutic strategies aimed at restoring microbial homeostasis to prevent or mitigate these conditions and promote longevity, the focus of the subsequent section.

**Figure 4:**
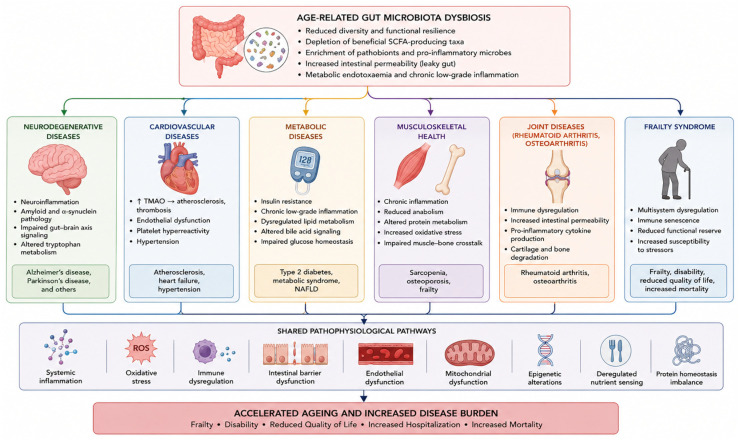
Gut microbiota involvement in age-related diseases. SCAs: short-chain fatty acids; TMAO: trimethylamine N-oxide; NAFLD: non-alcoholic fatty liver disease; ROS: reactive oxygen species.

Figure 4 summarises the major age-related diseases associated with gut microbiota alterations and highlights the principal microbiota-dependent mechanisms implicated in disease pathophysiology.

Table 1 summarises the principal age-related conditions currently associated with gut microbiota alterations, the main mechanistic pathways involved, representative findings from the literature, and the overall strength and limitations of the available evidence.

## 5. Longevity-Promoting Strategies Targeting the Gut Microbiota

The accumulating evidence that age-related gut microbiota dysbiosis contributes to inflammaging, accelerates multiple hallmarks of ageing, and exacerbates prevalent age-related diseases has naturally positioned the intestinal microbial ecosystem as a promising therapeutic target for extending healthspan and potentially lifespan [14,15,21]. Unlike many anti-ageing interventions that remain experimental, microbiota-targeted approaches benefit from being modifiable through accessible lifestyle and dietary means, while also offering more invasive or pharmacological options for higher-risk individuals. This section provides an extensive, critical synthesis of the current evidence base for longevity-promoting strategies that act primarily or partially through remodelling of the gut microbiota. It draws exclusively upon real peer-reviewed empirical studies, randomised controlled trials, longitudinal cohorts, and mechanistic investigations published in high-impact journals. Strategies are organised from the most scalable and evidence-based (dietary patterns and lifestyle) to targeted microbial therapeutics (prebiotics, probiotics, synbiotics, postbiotics, and faecal microbiota transplantation) and finally to pharmacological and emerging precision approaches. Particular attention is paid to interventions tested in older adults (≥65 years), their impact on microbial composition and function, downstream effects on inflammatory markers, frailty indices, metabolic health, and cognitive function, and the extent to which they may mimic the microbial configurations observed in centenarians and individuals with healthy ageing trajectories [12,14].

To facilitate comparison across the major microbiota-targeted longevity interventions currently investigated in older adults, Table 2 summarises the principal therapeutic strategies, their proposed mechanisms of action, representative findings from human and translational studies, the current strength of evidence, and key supporting references. Collectively, these interventions aim to restore microbial diversity, enhance short-chain fatty acid production, reduce inflammaging, and promote physiological resilience during ageing.

### 5.1. Dietary Patterns, Fibre Intake, and Lifestyle Modulation

Dietary modulation represents the most robust, cost-effective, and translatable strategy. Long-term adherence to diets rich in diverse plant fibres, polyphenols, and fermented foods consistently enhances microbial alpha diversity, enriches SCFA-producing taxa (*Faecalibacterium*, *Roseburia*, *Eubacterium*, *Bifidobacterium*), increases butyrate and propionate production, strengthens intestinal barrier integrity, and attenuates inflammaging [4,29]. The landmark NU-AGE trial remains the most compelling demonstration in older adults. Ghosh et al. [15] conducted a one-year Mediterranean diet intervention across 612 community-dwelling individuals aged 65–79 years in five European countries. The personalised Mediterranean diet, rich in legumes, whole grains, fruits, vegetables, nuts, olive oil, and moderate fermented foods, produced significant increases in microbial diversity and specific fibre-degrading taxa, elevated SCFA levels, reduced pro-inflammatory cytokines (IL-6, CRP), and improved frailty scores and cognitive function compared with control diets. Crucially, mediation analysis demonstrated that a substantial proportion of the health benefits regarding inflammaging and physical function were statistically attributable to microbiota remodelling. These findings align with earlier observational work showing that individuals with higher adherence to Mediterranean or plant-based dietary patterns exhibit microbial profiles resembling those of younger adults and long-lived cohorts, with enhanced capacity for polysaccharide degradation and secondary bile acid metabolism [12,29]. Several interventional studies in older adults have reported beneficial microbiota-related effects with daily dietary fibre intakes ranging approximately from 25 to 35 g/day, primarily through increased consumption of legumes, whole grains, vegetables, fruits, and resistant starch sources. Mediterranean dietary interventions such as the NU-AGE study generally promoted fibre intakes near or above current European nutritional recommendations for older adults, which are frequently unmet in Western populations [4,15].

Polyphenol-rich foods (berries, cocoa, green tea, red wine in moderation) exert prebiotic-like effects by selectively stimulating *Akkermansia muciniphila* and butyrate producers while inhibiting pathobionts [4]. Similarly, diets high in resistant starch and non-digestible oligosaccharides provide substrate for saccharolytic fermentation, counteracting the fibre-depletion typical of Westernised elderly diets [8]. Hydration status may also contribute to the maintenance of gastrointestinal and microbial homeostasis during ageing. Adequate fluid intake supports intestinal motility, facilitates nutrient transit, and helps preserve the integrity of the intestinal mucus layer, thereby promoting a favourable environment for host–microbiota interactions. Furthermore, sufficient hydration may reduce the risk of constipation, a highly prevalent condition among older adults that has been associated with alterations in gut microbial composition and metabolic activity. Although the direct effects of hydration on gut microbiota structure remain less extensively investigated than those of dietary patterns and fibre intake, emerging evidence suggests that adequate water consumption may indirectly support microbial diversity, gastrointestinal function, and overall gut health [52,53]. Therefore, hydration should be considered alongside dietary quality as part of a comprehensive lifestyle strategy to promote healthy ageing and maintain gut homeostasis. Longitudinal data from the NuAge cohort in Canada and European studies further indicate that sustained dietary quality in later life can partially reverse age-related losses in microbial richness and is associated with lower all-cause mortality risk, independent of other lifestyle factors [14]. However, responsiveness is highly individual; “non-responders” often harbour baseline microbiota with low diversity or specific enterotypes less equipped to metabolise the introduced substrates, highlighting the need for personalised nutrition [4].

### 5.2. Physical Activity and Exercise-Related Microbiota Modulation

Physical activity and exercise training constitute another accessible longevity strategy with documented microbiota-mediated benefits. Regular moderate aerobic and resistance exercise in older adults has been associated with modulation of gut microbiota composition and functional potential, including enrichment of short-chain fatty acid (SCFA)-producing taxa and improvements in host metabolic and inflammatory profiles [31,32]. Emerging evidence suggests that these microbiota-related changes may extend to broader physiological outcomes relevant to aging, including muscle function and cognitive performance, consistent with a putative gut–muscle–brain axis [7,27]. Mechanistically, exercise-related increases in microbial production of metabolites such as butyrate have been proposed to influence host energy metabolism and anti-inflammatory signalling pathways, although causality in humans remains to be fully established. Combined diet and exercise interventions may exert synergistic effects on gut microbiota composition and metabolic outputs [15].

### 5.3. Prebiotics, Probiotics, Synbiotics, and Postbiotics

Targeted microbial supplementation strategies (prebiotics, probiotics, and synbiotics) have been extensively tested in older populations, albeit with heterogeneous outcomes. Prebiotics such as fructo-oligosaccharides (FOS), galacto-oligosaccharides (GOS), and inulin selectively stimulate Bifidobacterium and Lactobacillus species, increase SCFA output, improve stool consistency, and modestly reduce inflammatory markers in institutionalised and community-dwelling elderly [21,44]. Randomised trials lasting 8–24 weeks have reported improvements in frailty indices and reduced incidence of infections, although effects on hard longevity endpoints remain unproven. Probiotic interventions using specific strains (Bifidobacterium longum, Bifidobacterium bifidum, Lactobacillus rhamnosus, Lactobacillus plantarum) have shown promise in restoring barrier function, decreasing LPS translocation, and modulating the microbiota–gut–brain axis to improve mood and cognitive parameters in mild cognitive impairment [7]. Synbiotic combinations (prebiotic plus probiotic) often yield superior results, with some trials reporting sustained increases in beneficial taxa and reductions in frailty scores over 12–24 weeks [5]. Recent translational studies have highlighted the growing potential of microbiota-targeted interventions for promoting healthy ageing, while also emphasizing the need for standardized protocols, strain-specific evaluation, and long-term clinical outcome assessments [42,45].

### 5.4. Faecal Microbiota Transplantation and Next-Generation Therapies

Next-generation approaches include the administration of *Akkermansia muciniphila* (live or pasteurised) and postbiotics (microbial metabolites such as butyrate capsules or cell-wall components). Early-phase human trials in metabolic syndrome and older adults suggest these can improve insulin sensitivity, reduce inflammatory tone, and strengthen mucosal integrity without the viability issues associated with live probiotics [21]. Faecal microbiota transplantation (FMT) offers the most direct method of ecosystem restoration. While primarily validated for recurrent *Clostridioides difficile* infection (where elderly patients represent the highest-risk group and achieve cure rates exceeding 90%), emerging exploratory trials have tested FMT for frailty, metabolic syndrome, and neurodegenerative conditions [30]. Small proof-of-concept studies in older adults have reported increased microbial diversity, enrichment of SCFA producers, reduced inflammaging markers, and modest improvements in gait speed and cognitive scores post-FMT from young healthy donors. However, safety concerns (particularly bacteraemia risk in frail immunocompromised hosts), standardisation of donor selection, and long-term durability remain significant barriers. The microbial signatures of exceptionally long-lived individuals (enriched *Akkermansia*, Christensenellaceae, and butyrate pathways) provide a rational template for “ideal” donor material or engineered consortia [12,14].

### 5.5. Pharmacological and Precision-Medicine Approaches

Pharmacological agents with established or putative anti-ageing properties increasingly demonstrate microbiota-dependent mechanisms. Metformin, widely used in type 2 diabetes and under investigation in the TAME trial for ageing, remodels the gut microbiota by increasing *Akkermansia* and SCFA producers while decreasing pathobionts; some of its insulin-sensitising and anti-inflammatory effects appear mediated by these changes [33]. Rapamycin and its analogues (rapalogs) influence microbial ecology in model organisms, although human data in older adults are limited. Senolytic compounds and mTOR inhibitors may indirectly benefit the microbiota by reducing SASP-driven alterations in the intestinal niche [17]. These pleiotropic effects underscore the bidirectional interactions between host-targeted drugs and the microbiome.

Emerging precision strategies leverage multi-omics profiling (metagenomics, metabolomics, host epigenomics) to tailor interventions. Machine-learning models trained on large cohorts can now predict individual responses to dietary fibre or specific probiotics with reasonable accuracy, opening the door to personalised microbiome-based longevity programmes [4,14]. Synthetic biology approaches, including engineered bacteria that produce anti-inflammatory compounds or degrade pro-ageing metabolites (e.g., TMA), are in preclinical development. Longitudinal monitoring of the microbiome as a biological age clock itself (using deviations from “healthy ageing” configurations) may enable early intervention before clinical frailty manifests [14].

### 5.6. Current Limitations and Translational Challenges

Despite these promising data, the evidence base has important limitations that temper enthusiasm. Most probiotic and prebiotic trials in older adults are small, short-duration (<6 months), and rely on surrogate endpoints (inflammatory biomarkers, frailty scales) rather than disability-free survival or lifespan. Effect sizes are often modest and highly heterogeneous due to baseline microbiota composition, concurrent polypharmacy, and geographic variation [8,39]. FMT, while dramatic in *C. difficile* treatment, lacks large-scale randomised data for longevity indications, and regulatory frameworks remain evolving. Causality is difficult to disentangle; improvements in health may themselves reshape the microbiota. Moreover, interventions that work well in young or middle-aged adults frequently show blunted efficacy in the oldest-old because of immunosenescence and altered host–microbe crosstalk [9,10].

### 5.7. Concluding Statements

Collectively, these findings suggest that microbiota-targeted interventions may represent a complementary component of multidimensional healthy ageing strategies rather than stand-alone anti-ageing therapies.

**Figure 5:**
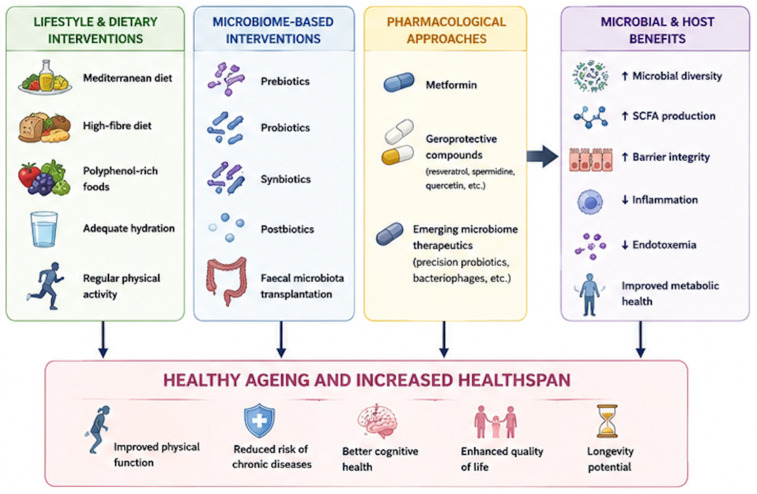
Microbiota-targeted interventions for healthy ageing.

Nevertheless, the convergence of mechanistic, observational, and interventional evidence supports the conclusion that maintenance or restoration of a diverse, SCFA-rich, anti-inflammatory gut microbiota configuration is a realistic and actionable component of healthy longevity strategies. By integrating dietary patterns such as the Mediterranean diet, regular physical activity, judicious use of targeted microbial supplements, and, where appropriate, more invasive approaches, clinicians and researchers may meaningfully influence ageing trajectories. These microbiota-targeted interventions appear particularly effective when embedded within a multidimensional geroscience framework that also addresses nutrition, exercise, sleep, and social connection. While this review centres on biological mechanisms linking gut microbiota to inflammaging and age-related diseases, complementary perspectives underscore the psychological and existential layers of ageing: existential boredom as a marker of subjective anomie and disconnection [54], the intersections of boredom, faith, and hope in the human condition [55], the importance of proactivity for well-being [56], and relational models of consciousness and multispecies responsibility essential for planetary health [57,58]. Current and emerging microbiota-targeted strategies that may support healthy ageing and promote healthspan are summarised in Figure 5.

The final section of this review will critically examine the methodological challenges, unresolved questions, and future research priorities necessary to translate this rapidly evolving field into evidence-based clinical practice.

## 6. Challenges, Limitations, Future Directions, and Conclusions

### 6.1. Methodological and Longitudinal Challenges

Despite the substantial progress in elucidating the bidirectional relationship between the gut microbiota and human ageing, significant challenges and limitations continue to constrain the translation of this knowledge into reliable clinical applications for promoting longevity and mitigating age-related diseases. These limitations span methodological, biological, ethical, and translational domains and must be rigorously addressed if the gut microbiome is to fulfil its promise as both a biomarker of biological age and a modifiable therapeutic target [8,14,39]. This final section offers an extensive critical examination of these challenges, delineates priority areas for future research, and synthesises the overarching narrative of the review, arguing that while the gut microbiota represents a compelling nexus in geroscience, its successful targeting will require unprecedented interdisciplinary integration, methodological refinement, and personalised approaches grounded in robust causal evidence.

A primary methodological challenge lies in the predominance of cross-sectional study designs. The majority of human microbiome–ageing research, including many seminal contributions, compares young versus old cohorts at a single time point, rendering it impossible to distinguish whether observed microbial shifts are causes, consequences, or merely correlates of ageing trajectories [10,11,12]. Longitudinal studies that repeatedly sample the same individuals across decades remain scarce because of high costs, participant attrition, and the logistical difficulties of maintaining consistent sampling protocols over years. Those that do exist, such as the work by Jeffery et al. [13] and Wilmanski et al. [14], reveal highly individualised microbial trajectories, with some older adults maintaining “youthful” configurations while others experience rapid destabilisation linked to hospitalisation, antibiotic exposure, or acute illness. This heterogeneity underscores that chronological age alone is a poor predictor of microbiota status; frailty, comorbidity burden, diet, and medication use exert stronger influences [27,28].

### 6.2. Confounding Variables and Population Heterogeneity

Confounding factors further complicate interpretation. Older adults are subject to extensive polypharmacy, with proton-pump inhibitors, antibiotics, non-steroidal anti-inflammatory drugs, statins, and metformin all exerting profound, sometimes lasting effects on microbial composition and function [21,33]. Diet is another critical confounder; institutionalised elderly individuals often consume low-fibre, nutrient-poor diets that independently drive dysbiosis, making it difficult to isolate age-specific effects [15,29]. Geographic, ethnic, and socioeconomic variation adds additional layers of complexity. Microbial signatures documented in European or North American cohorts are not always replicated in Asian, African, or indigenous populations, where lifelong dietary patterns, sanitation, and pathogen exposure differ markedly [16,34]. Host genetics, including polymorphisms in genes influencing mucus production (FUT2 secretor status) or immune recognition of microbial patterns, further modulate responsiveness and must be integrated into future models [5].

### 6.3. Technical, Analytical, and Multi-Omics Limitations

Technical and analytical limitations are equally pressing. Faecal sampling, the mainstay of most studies, predominantly captures luminal rather than mucosa-associated communities that interact most intimately with the host epithelium and immune system [44]. Variations in DNA extraction kits, 16S rRNA hypervariable regions sequenced, sequencing platforms, and bioinformatics pipelines (QIIME2, DADA2, Kraken, MetaPhlAn) introduce substantial batch effects and reduce reproducibility across laboratories [39]. Functional inference from taxonomic data remains imperfect; shotgun metagenomics and metabolomics provide deeper insights but are more expensive and still struggle to differentiate microbial from host-derived metabolites [4]. Moreover, most studies focus on bacteria while under-representing archaea, fungi, viruses, and bacteriophages, whose roles in inflammaging and longevity may be substantial [7].

### 6.4. Challenges in Establishing Causality and Translational Safety

Establishing causality remains the central scientific limitation. While gnotobiotic mouse models and faecal microbiota transplantation (FMT) experiments have provided elegant mechanistic demonstrations—such as the reversal of intestinal permeability and macrophage dysfunction by young microbiota in aged mice [9]—rodent microbiomes and immune systems differ fundamentally from those of humans. Human FMT trials for non-*Clostridioides difficile* indications are still small, often uncontrolled, and raise important safety concerns regarding pathogen transmission, particularly in frail, immunocompromised older recipients [30]. Ethical considerations surrounding donor selection, long-term microbial engraftment, and equitable access to advanced microbiome therapies further constrain progress.

### 6.5. Limitations of Current Intervention Studies

Intervention trials targeting the microbiota for longevity endpoints face additional hurdles. Most probiotic, prebiotic, and synbiotic studies in older adults are short-term (4–24 weeks), underpowered, and rely on surrogate biomarkers (CRP, IL-6, faecal calprotectin, frailty indices) rather than hard clinical outcomes such as disability-free survival, incidence of multimorbidity, or all-cause mortality [5,21]. Effect sizes are frequently modest and display high inter-individual variability, suggesting that “one-size-fits-all” approaches are inadequate. The Mediterranean diet intervention in the NU-AGE trial [15] represents a notable exception in scale and duration, yet even here mediation by the microbiota, while statistically significant, accounted for only a portion of the observed health benefits, indicating multifactorial mechanisms.

### 6.6. Future Directions: Multi-Omics, AI, and Precision Geroscience

These limitations notwithstanding, the field is poised for transformative advances. Future research must prioritise large-scale, multi-centre longitudinal cohorts that integrate deep multi-omics profiling (metagenomics, metatranscriptomics, metaproteomics, and metabolomics) with host epigenomics, immunophenotyping, clinical phenotyping, and wearable sensor data. Such integrated “microbiome–host–exposome” atlases, ideally spanning from mid-life into extreme old age, would enable the development of robust microbial clocks of biological age that complement existing epigenetic and proteomic clocks [14,20]. Machine-learning and artificial intelligence approaches will be essential for identifying predictive microbial consortia, personalising dietary and therapeutic interventions, and stratifying older adults according to their microbiome “ageing type” (responders versus non-responders). Recent systems-biology studies have further demonstrated that distinct intestinal microbial signatures are associated with accelerated systemic and intestinal biological ageing, reinforcing the translational potential of integrating metagenomics, metabolomics, and artificial intelligence-driven predictive modelling to identify microbiome-based biomarkers of healthy ageing and guide precision geroscience interventions [59].

### 6.7. Future Therapeutic and Experimental Approaches

Causal inference must be strengthened through well-designed human intervention studies. Randomised controlled trials of microbiota-targeted therapies should adopt longer durations (≥2 years), larger sample sizes (>500 participants), and clinically meaningful primary endpoints such as frailty reversal, delay in onset of multimorbidity, or improvement in healthspan metrics. Hybrid designs combining Mediterranean diet interventions with stratified probiotic or synbiotic supplementation, guided by baseline microbiome profiling, represent a promising avenue [4,15]. Next-generation therapeutics—including defined microbial consortia engineered from signatures observed in centenarians (*Akkermansia*, Christensenellaceae, specific butyrate producers), postbiotics, and live biotherapeutic products—require rigorous phase II/III testing with stringent safety monitoring in geriatric populations [12,30]. In parallel, healthy ageing is influenced not only by biological and microbiota-related factors but also by broader psychosocial determinants. Consequently, low-cost and scalable interventions such as social prescribing deserve greater attention in future research. Ferreira et al. [60] demonstrated that social prescribing programmes significantly improved self-esteem in older adults (*p* < 0.001), with a positive trend in cognitive performance, reinforcing the value of holistic approaches that target the social determinants of ageing.

### 6.8. Mechanistic Research Priorities Beyond Bacterial Taxonomy

Mechanistic research should expand beyond traditional germ-free and FMT models to include humanised gnotobiotic mice, organ-on-chip systems, and human intestinal organoids colonised with aged microbiota. Particular emphasis should be placed on understanding how microbial signals interact with the expanded hallmarks of ageing, including disabled autophagy, senescence-associated secretory phenotype propagation, and compromised extracellular matrix remodelling [17,20]. The role of the virome, mycobiome, and microbial extracellular vesicles in modulating inflammaging and the microbiota–gut–brain axis warrants dedicated investigation [7].

### 6.9. Translational Implementation, Public Health, and Equity

Translational and implementation science must address equity. Interventions shown to be effective in high-income settings need validation in low- and middle-income countries where dietary transitions, antibiotic overuse, and ageing demographics intersect differently. Cost-effectiveness analyses, regulatory frameworks for microbiome-based therapies, and guidelines for clinicians on when and how to target the gut microbiota in older patients will be required for widespread adoption. Public health strategies that promote lifelong dietary quality, physical activity, and prudent antibiotic stewardship may prove the most scalable means of preserving microbial homeostasis into late life [3,4].

### 6.10. General Conclusions

In conclusion, this narrative review has synthesised a substantial body of peer-reviewed evidence demonstrating that the gut microbiota undergoes progressive, context-dependent remodelling with advancing age, characterised by reduced taxonomic and functional diversity, depletion of SCFA-producing taxa, and relative expansion of pathobionts. These changes are increasingly recognised as likely contributors to inflammaging and several hallmarks of ageing, although causal relationships remain incompletely established in humans and likely promote or exacerbate prevalent age-related diseases including neurodegenerative disorders, cardiovascular disease, type 2 diabetes, sarcopenia, osteoporosis, and frailty [7,9,10,14,15]. Conversely, the distinct microbial configurations observed in centenarians and individuals exhibiting healthy ageing trajectories suggest that maintenance of specific metabolic functionalities (particularly robust SCFA and secondary bile acid pathways) may constitute a feature of successful longevity rather than mere survival bias [12,16].

Evidence-based strategies targeting the microbiota, ranging from Mediterranean-style dietary patterns and exercise to precision probiotics, synbiotics, postbiotics, and carefully screened FMT, show genuine potential to restore microbial homeostasis, attenuate inflammaging, improve clinical phenotypes, and extend healthspan [4,21,30]. Nevertheless, substantial methodological, causal, and translational gaps remain. Overcoming these will require concerted investment in longitudinal multi-omics cohorts, rigorously designed personalised intervention trials, advanced experimental models, and equitable implementation frameworks.

Ultimately, the gut microbiota should be viewed not as a separate entity but as an integral component of the ageing human superorganism. By nurturing microbial ecology throughout life and deploying targeted restoration strategies in later decades, it may become possible to compress morbidity, preserve functional independence, and enable more individuals to reach extreme old age in good health. While the journey from associative observation to causal, personalised, clinically validated interventions remains incomplete, the trajectory is clear: microbiome research represents one of the most promising and rapidly evolving areas within contemporary geroscience. Realising its full potential for human longevity will demand the same rigorous, collaborative, and innovative spirit that has characterised the field’s rapid evolution since the advent of high-throughput sequencing. The next generation of geroscience will increasingly depend on integrating microbial ecology with complementary molecular regulatory systems governing the ageing process, including epigenetic, metabolic, immunological, and post-transcriptional mechanisms. Such multidimensional approaches have the potential to transform microbiome research from a predominantly associative discipline into a mechanistically grounded framework capable of supporting personalised interventions for healthy ageing and longevity [61].

## Figures and Tables

**Table 1:** Evidence Linking Gut Microbiota Alterations to Major Age-Related Diseases.

Disease/Condition	Main Microbiota-Related Mechanisms	Representative Findings	Strength of Current Evidence	Main Limitations	Key References
Alzheimer’s disease	Reduced SCFA production, increased intestinal permeability, neuroinflammation, altered gut–brain signalling	Reduced microbial diversity and increased pro-inflammatory taxa have been associated with amyloid pathology and cognitive decline; germ-free and antibiotic-treated mouse models demonstrate modulation of amyloid deposition and microglial activation	Moderate	Predominantly associative human studies; limited longitudinal and causal evidence	[23,24,46]
Parkinson’s disease	α-synuclein aggregation, altered vagal signalling, SCFA dysregulation, neuroimmune activation	Reduced SCFA-producing taxa and increased Proteobacteria frequently reported; microbiota transplantation from Parkinson’s disease patients worsened neuroinflammation and motor dysfunction in animal models	Moderate-to-strong	Human studies remain heterogeneous; limited large longitudinal cohorts	[7,22]
Cardiovascular disease	TMAO production, endothelial dysfunction, systemic inflammation, altered bile acid metabolism	Elevated circulating TMAO associated with atherosclerosis and adverse cardiovascular outcomes; microbial metabolism of dietary choline and carnitine implicated mechanistically	Strong	Dietary confounding, medication effects, and inter-individual variability	[25,26]
Type 2 diabetes mellitus	Metabolic endotoxaemia, impaired SCFA signalling, insulin resistance, chronic low-grade inflammation	Reduced butyrate-producing taxa and altered microbial metabolic pathways consistently reported in individuals with metabolic dysfunction	Strong	Bidirectional host–microbiome interactions complicate causality	[47,48]
Frailty and sarcopenia	Inflammaging, mitochondrial dysfunction, anabolic resistance, impaired gut–muscle axis	Frail older adults frequently exhibit lower microbial diversity and reduced SCFA-producing taxa associated with poorer physical performance	Moderate	Limited intervention studies and variable frailty definitions	[27,28]
Osteoporosis	Altered calcium absorption, immune-mediated osteoclast activation, chronic inflammation	Dysbiosis-associated inflammation and reduced SCFA production linked to lower bone mineral density and impaired bone metabolism	Moderate	Limited longitudinal human evidence	[44,49]
Rheumatoid arthritis and osteoarthritis	Increased intestinal permeability, immune dysregulation, chronic low-grade inflammation, altered SCFA production, gut–joint axis	Gut microbiota dysbiosis has been associated with increased inflammatory activity, cartilage degradation, altered immune tolerance, and disease severity. Experimental and clinical studies suggest that microbial metabolites and intestinal barrier dysfunction may contribute to both rheumatoid arthritis and osteoarthritis progression.	Moderate	Limited longitudinal studies; causality remains incompletely established; substantial heterogeneity between cohorts	[49,50,51]
Chronic kidney disease	Increased uraemic toxin production, systemic inflammation, gut–kidney axis dysregulation	Altered microbiota may contribute to increased production of indoxyl sulphate and p-cresyl sulphate	Moderate	Significant confounding by comorbidities and medication use	[4,14]
Healthy ageing/longevity	Maintenance of microbial diversity, preserved SCFA and secondary bile acid pathways, reduced inflammaging	Centenarians frequently exhibit distinct microbial configurations associated with lower inflammatory burden	Moderate	Lack of definitive causal human evidence	[12,14]

SCFA = short-chain fatty acid; TMAO = trimethylamine N-oxide. Strength of evidence was qualitatively evaluated by the authors based on consistency of observational findings, availability of mechanistic evidence, longitudinal human data, and translational/interventional support. The table synthesises representative findings from the cited literature and does not constitute a formal systematic evidence grading approach.

**Table 2:** Microbiota-Targeted Longevity Interventions in Older Adults.

Intervention	Proposed Mechanism	Main Findings	Evidence Strength	Key References
Mediterranean diet	Increases microbial diversity, SCFA production, and fibre-degrading taxa; reduces inflammaging	Improved frailty scores, cognitive function, inflammatory markers, and microbial diversity in older adults	Strong	[4,15,29]
Physical exercise	Enhances SCFA-producing taxa, metabolic regulation, and anti-inflammatory signalling	Associated with improved metabolic and inflammatory profiles and enrichment of beneficial taxa	Moderate	[27,31,32]
Prebiotics	Selectively stimulate beneficial bacteria (e.g., Bifidobacterium, Lactobacillus) and SCFA synthesis	Improved stool consistency, inflammatory markers, and some frailty-related outcomes	Moderate	[21,44]
Probiotics	Restore microbial balance, strengthen barrier integrity, and modulate immune responses	Improvements reported in barrier function, mood, cognition, and inflammatory profiles	Moderate	[7,21]
Synbiotics	Combined prebiotic and probiotic effects enhance microbial engraftment and SCFA production	Some trials demonstrate reductions in frailty scores and sustained increases in beneficial taxa	Moderate	[5,21]
Postbiotics	Deliver microbial metabolites or cell-wall products directly without live organisms	Early evidence suggests improved insulin sensitivity and mucosal integrity	Emerging	[21]
Faecal microbiota transplantation (FMT)	Restores microbial ecosystem diversity through donor microbiota transfer	Increased microbial diversity and preliminary improvements in inflammatory and frailty-related markers	Emerging-to-moderate	[12,30]
Metformin	Remodels microbiota towards increased Akkermansia and SCFA-producing taxa	Some anti-inflammatory and metabolic benefits may be microbiota mediated	Moderate	[33]
Precision microbiome approaches	Use multi-omics and machine learning to personalise interventions	Potential to predict responder profiles and optimise microbiota-targeted therapies	Emerging	[4,14]

SCFA = short-chain fatty acid; FMT = faecal microbiota transplantation. Evidence strength was qualitatively evaluated based on consistency of findings, mechanistic support, translational evidence, and availability of human interventional data.

## Data Availability

No new data were created or analysed in this study.
